# How to Assess the Impact of Catheter Ablation for Atrial Fibrillation in Patients With Heart Failure With Preserved Ejection Fraction

**DOI:** 10.1002/clc.70231

**Published:** 2025-12-02

**Authors:** Naoya Kataoka, Teruhiko Imamura

**Affiliations:** ^1^ Second Department of Internal Medicine University of Toyama Toyama Japan

To the Editor,

The prognostic impact of catheter ablation in patients with atrial fibrillation (AF) and heart failure with preserved ejection fraction (HFpEF) remains controversial. Using a nationwide database, the authors demonstrated that catheter ablation was associated with cardiovascular and cerebral benefits, as well as a reduction in all‐cause mortality, in patients with AF and HFpEF [1]. Several concerns arise from these findings.

The authors assessed all‐cause mortality in the HFpEF cohort, although the prescription rates of guideline‐directed medical therapy—including sodium–glucose cotransporter‐2 inhibitors—were extremely low [1]. This may limit the interpretation of mortality reduction attributable to catheter ablation itself.

The major causes of death in HFpEF are generally noncardiac [2]. The mechanism by which catheter ablation would reduce all‐cause mortality in this population therefore remains unclear. Catheter ablation helps prevent thrombus formation and stroke, and may reduce worsening heart failure by preserving atrial contraction. However, it is unlikely to directly decrease noncardiac events, which represent a substantial proportion of deaths in HFpEF.

The authors excluded patients who underwent valve replacement [1]. Yet, one of the most advanced clinical scenarios of HFpEF with AF involves significant valvular disease—particularly mitral regurgitation—that requires intervention [3]. Including such patients would improve the generalizability of the findings to a more representative HFpEF population. Moreover, individuals with severe valvular disease often have markedly remodeled atria and are seldom considered for catheter ablation.

Another important issue not evaluated in this study is bleeding related to anticoagulant therapy [1]. Patients with HFpEF may be at higher risk of bleeding complications than those with reduced ejection fraction [4]. In this regard, we strongly recommend considering left atrial appendage closure at the time of catheter ablation to mitigate anticoagulant‐related bleeding risk, if applicable.

## Ethics Statement

The authors have nothing to report.

## Consent

The authors have nothing to report.

## Conflicts of Interest

The authors declare no conflicts of interest.

## Data Availability

The authors have nothing to report.
